# General practice-based cancer research publications: a bibliometric analysis 2013–2019

**DOI:** 10.3399/BJGP.2022.0025

**Published:** 2022-12-13

**Authors:** Kristi Milley, Sophie Chima, Napin Karnchanachari, Mairead McNamara, Paige Druce, Jon Emery

**Affiliations:** Herman professor of primary care cancer research, Centre for Cancer Research and Department of General Practice, Victorian Comprehensive Cancer Centre, University of Melbourne, Melbourne.; Herman professor of primary care cancer research, Centre for Cancer Research and Department of General Practice, Victorian Comprehensive Cancer Centre, University of Melbourne, Melbourne.; Herman professor of primary care cancer research, Centre for Cancer Research and Department of General Practice, Victorian Comprehensive Cancer Centre, University of Melbourne, Melbourne.; Department of Cancer Imaging, Peter MacCallum Cancer Centre, Parkville.; Central Clinical School, Monash University, Melbourne.; Herman professor of primary care cancer research, Centre for Cancer Research and Department of General Practice, Victorian Comprehensive Cancer Centre, University of Melbourne, Melbourne.

**Keywords:** bibliometrics, cancer, general practice, primary health care

## Abstract

**Background:**

General practice plays a critical role in the prevention, diagnosis, management, and survivorship care of patients with cancer. Mapping research outputs over time provides valuable insights into the evolving role of general practice in cancer care.

**Aim:**

To describe and compare the distribution of cancer in general practice research publications by country, cancer type, area of the cancer continuum, author sex, and journal impact factor.

**Design and setting:**

A bibliometric analysis using a systematic approach to identify publications.

**Method:**

MEDLINE and Embase databases were searched for studies published between 2013 and 2019, which reported on cancer in general practice. Included studies were mapped to the cancer continuum framework. Descriptive statistics were used to present data from the included studies.

**Results:**

A total of 2798 publications were included from 714 journals, spanning 79 countries. The publication rate remained stable over this period. Overall, the US produced the most publications (*n* = 886, 31.7%), although, per general population capita, Denmark produced nearly 10 times more publications than the US (20.0 publications per million compared with 2.7 publications per million). Research across the cancer continuum varied by country, but, overall, most studies focused on cancer screening, diagnosis, and survivorship. More than half of included studies used observational study designs (*n* = 1523, 54.4%). Females made up 66.5% (*n* = 1304) of first authors, but only 47.0% (*n* = 927) of last authors.

**Conclusion:**

Cancer in general practice is a stable field where research is predominantly observational. There is geographical variation in the focus of cancer in general practice research, which may reflect different priorities and levels of investment between countries. Overall, these results support future consideration of how to improve under-represented research areas and the design, conduct, and translation of interventional research.

## INTRODUCTION

In 2020, there were an estimated 19.3 million new cancer diagnoses globally resulting in nearly 10 million cancer-related deaths.^1^ The role of general practice has continued to expand across the cancer continuum.^2^ Primary care is essential for the prevention and early detection of cancer.^2^^,^^3^ As the number of patients with cancer and survivors seen within general practice is predicted to double by 2040,^4^ the role GPs play in cancer survivorship is also evolving.^5^^,^^6^ The management of cancer is now frequently seen as the management of a chronic disease. Consequently, general practice has a critical role in the coordination of care, management of cancer and multimorbidity, as well as the secondary prevention of cancer.^6^

Cancer in primary care research is often cited as underfunded or neglected.^7^ Examining patterns of funding to eventual translation is important to understand research priorities in cancer and where gaps exist.

An approach to establishing research outputs in cancer in primary care research is through bibliometric analysis. Bibliometrics is a systematic approach to evaluate research outputs, which can help map changes in the interest and outputs of the research community over time.^8^ Bibliometric analysis has previously been used to investigate research output in primary care^9^ as well as in oncology settings.^10^^–^^12^ Bibliometrics has been used as a method to try to quantify cancer research funding.^13^^,^^14^ To date, there has not been an analysis of cancer research outputs in the general practice setting.

To better understand the primary care cancer research landscape, and more specifically general practice research, a bibliometric review was conducted. Research outputs from 2013–2019 were used to address the following research questions:

- Which countries contributed the most to cancer in general practice publications?
- How do countries compare when publications are mapped across the cancer continuum?
- What are the most common cancer types investigated in general practice and are any cancer types under-represented in the literature?
- Which study designs were most frequently used in cancer in general practice research?
- Which journals most commonly publish about cancer in general practice and what are their impact factors?
- What is the sex distribution of first and last authors in cancer in general practice research?
- Have publication rates changed over time?

**Table:** How this fits in

To the authors’ knowledge, this is the first study to explore and analyse cancer in general practice publications. It provides an overview of predominant study designs that are used in general practice research, where research is focused along the cancer continuum, and the prevalence of tumour type investigated in different countries.

## METHOD

This bibliometric analysis used a systematic approach to identify publications that presented or synthesised primary data of research studies that included both general practice and cancer. A systematic search strategy was used to ensure a robust method to collect the sample and a bibliometric review was then applied rather than undertaking a systematic review.

### Search strategy

An existing search, which was used in a previous Evidence Check published by the Sax Institute,^15^ was developed. This review focused on early detection through to follow-up and used the terms: cancer AND primary health care AND [diagnosis OR follow-up OR survivor]. This strategy was expanded to include the entire cancer continuum to include terms for prevention and palliative care. An original search was conducted in MEDLINE and Embase between January 2013 and December 2017 to identify studies from a 5-year period. A supplementary search was then conducted to include articles up to December 2019. The search strategy was broad and included word variations for ‘cancer’ and ‘primary care’, in addition to terms used across the cancer continuum (for example, prevention) (see Supplementary Figure S1).

### Study selection

Studies published in English, in which a component of the research question involved both general practice and cancer prevention, diagnosis, or cancer care, were included. As the terms ‘primary care’ and ‘general practice’ are often used interchangeably in many settings both were included along with their variations in the strategy, but publications were only included that focused on general practice. Narrative reviews, protocols, expert opinions (including editorials and commentaries), conference abstracts, and case studies were excluded. Studies where cancer was an incidental result and not related to the research question or outcome measures were also excluded. Using Covidence systematic review software, two researchers independently screened the search results, and a consensus approach with a third reviewer was adopted to resolve conflicts about eligibility.

### Cancer control continuum framework

The cancer continuum was used as a framework to categorise included studies.^3^ This well-established framework covers cancer control and care from prevention through to palliative care. The areas of the continuum used as categories were cancer risk, prevention, screening, diagnosis, treatment, palliative care, or cross-cutting.

### Data extraction and analysis

Included studies were exported from Covidence and the following information was extracted by one reviewer: study country; study design; journal impact factor; the area(s) of the cancer continuum; cancer type; and the sex of the first and last author. Given the sample size (*n* = 2798), a 10% cross-check of the data was performed by a second reviewer to ensure accuracy. Where appropriate, descriptive statistics, that is, frequency, mean, median, and range, were used to analyse and report on each variable.

### Geographical location

Publications that included more than one country either through study sites or author affiliations were categorised as ‘international’ collaborations. Countries were ranked by total number of publications; this was then divided by the country population (December 2019) to determine a per capita publication number. The calculation was based on population size (that is, number of publications per million people). This figure was used to identify the top 10 publishing countries per capita.

### Study design

Study design was categorised by experimental methodological approach (see Supplementary Table S1). Scoping and rapid reviews were included within systematic review study designs.

### Journals and impact factor

Journal titles were reviewed and adjusted for variations in abbreviations used by either database to ensure consistency. The website InCites (https://incites.clarivate.com) was used to identify the impact factor from the year of publication. If InCites did not reference the journal’s impact factor, Scimagojr (https://www.scimagojr.com) was used as a secondary source.

### Author sex

The author’s sex was established by reviewing LinkedIn, ResearchGate, or institutional affiliation staff pages. For analysis, sex was recorded from publications from 2015–2019. Single- author articles were included as first authors for analysis and, for the analysis of author sex, publications were excluded where author sex was unable to be identified.

### Publication rate

The relative change between years was calculated using:

$$(\mathrm{relative}\;\mathrm{change})C=\frac{(\mathrm{final}\;\mathrm{year})x_{2}-(\mathrm{initial}\;\mathrm{year})x_{1}}{(\mathrm{initial}\;\mathrm{year})x_{1}}$$

## RESULTS

**Figure 1.**
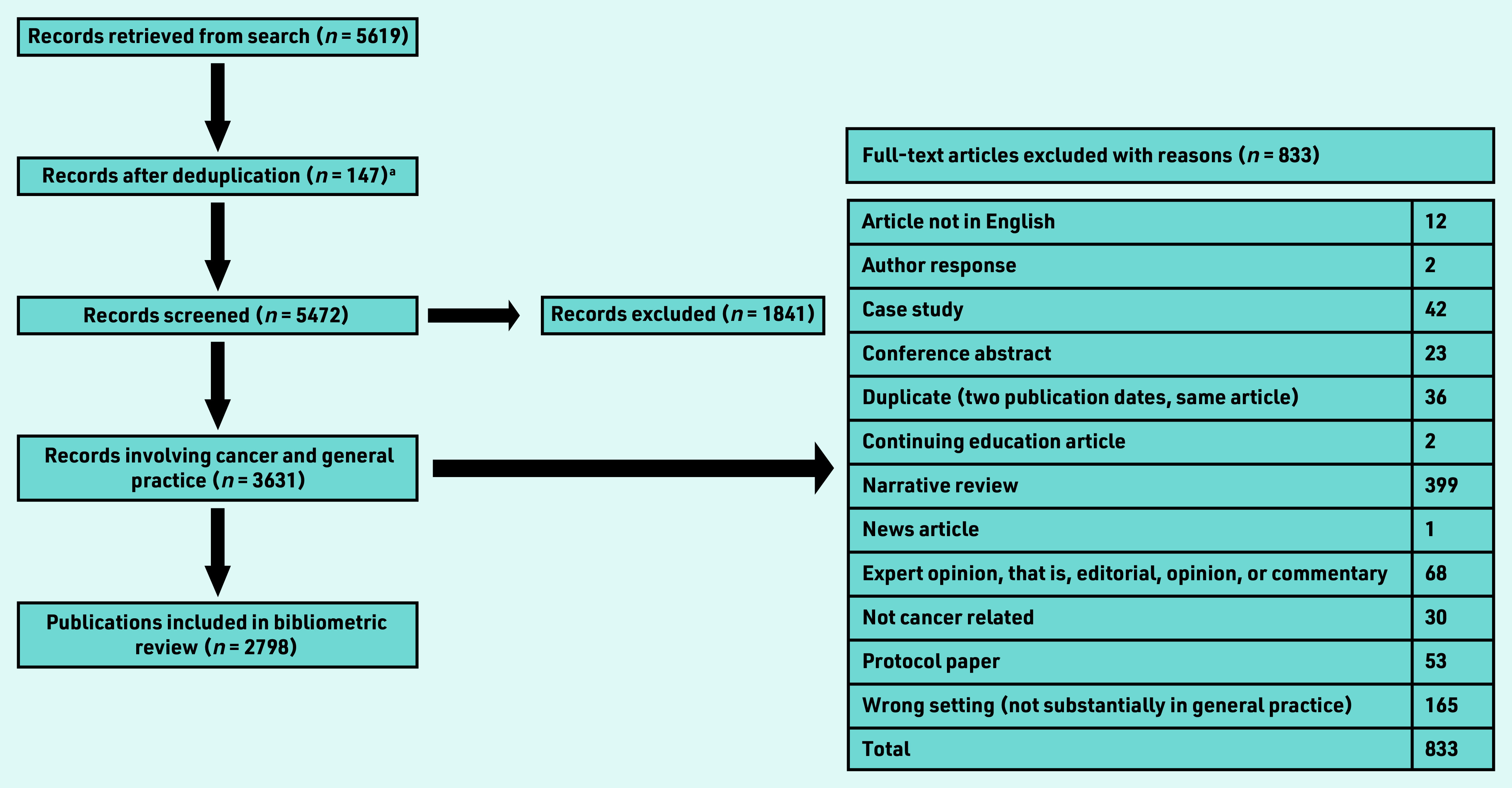
*Flowchart of publication selection process. aMEDLINE search excluded Embase records, reducing the number of duplicates. See search strategy for more details.*

Overall, 2798 articles were included for analysis (Figure 1). These were published in 714 journals with a median impact factor of 2.51; 75.0% (*n* = 1893/2524) of publications were published in journals with an impact factor of ≤3.5 (range 0–244.5). The impact factor was either not applicable, in the case of new journals, or not available for 274 publications. Publications from the US had the highest average journal impact factor (5.4), followed by the Netherlands (4.2) and the UK (4.1). Denmark, Canada, and Australia all published in lower impact journals with an average impact factor ranging between 2.5 and 2.8. The top 10 publishing journals were the *British Journal of General Practice* (*n* = 98), *BMJ Open* (*n* = 81), *PLoS One* (*n* = 70), *Journal of Cancer Education* (*n* = 63), *British Journal of Cancer* (*n* = 58), *Family Practice* (*n* = 54), *BMC Family Practice* (*n* = 52), *European Journal of Cancer Care* (*n* = 49), *BMC Cancer* (*n* = 48), *Preventive Medicine* (*n* = 43), and *Cancer* (*n* = 42). Together between 2013 and 2019 these top 10 journals had an average impact factor of 3.8 (data not shown).

Publications were produced in 79 countries. Table 1 outlines the top 10 countries based on overall publication number, and number of publications per capita. The US produced the most publications (*n* = 886, 31.7%), although, per general population capita, Denmark produced nearly 10 times more publications than the US (20.0 publications per million compared with 2.7 publications per million).

**Table 1.** Country publication rate by total publications and publications per capita

**Top 10 countries: total number of publications**			**Top 10 countries: number of publications per capita**		
**Rank**	**Country**	***n* (%)**	**Rank**	**Country**	**Publications per million**
**1**	US	886 (31.7)	**1**	Denmark	20.0
**2**	UK	543 (19.4)	**2**	UK	8.1
**3**	Australia	181 (6.5)	**3**	Netherlands	7.8
**4**	Canada	171 (6.1)	**4**	Australia	7.1
**5**	Netherlands	135 (4.8)	**5**	Canada	4.5
**6**	Denmark	116 (4.1)	**6**	US	2.7
**7**	Spain	67 (2.4)	**7**	Spain	1.4
**8**	France	66 (2.4)	**8**	France	1.0
**9**	Germany	46 (1.6)	**9**	Italy	0.7
**10**	Italy	39 (1.4)	**10**	Germany	0.6
**—**	International	99 (3.5)	**—**		
**—**	Remaining 69 countries	449 (16.0)	**—**		
	**Total**	**2798 (100)**			

**Figure 2.**
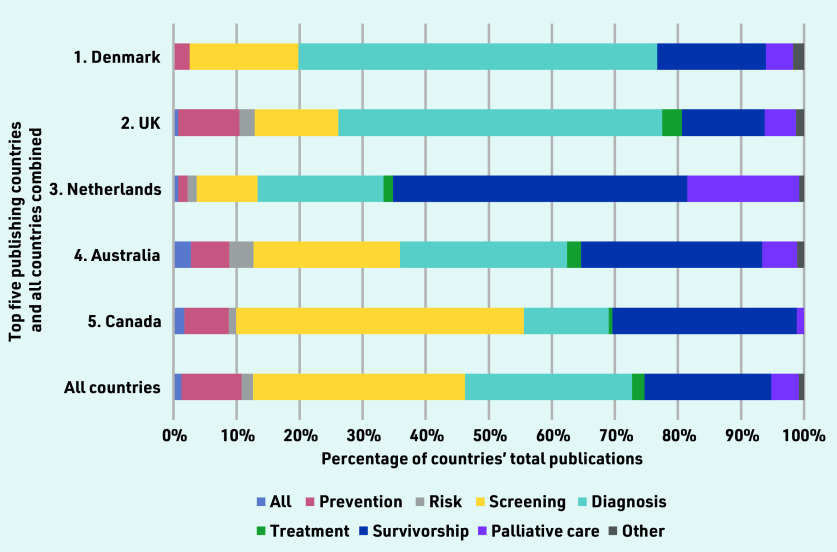
*Publications mapped across the cancer continuum for the five countries with the most publications per capita, compared with the overall distribution.*

When mapped across the cancer continuum, publications about cancer screening represented more than one- third of included studies (33.6%, *n* = 940), followed by diagnosis (26.5%, *n* = 742), and survivorship (20.1%, *n* = 563). Between-country comparisons showed that diagnosis was the most common area of research from the UK (51.4%, *n* = 279) and Denmark (56.9%, *n* = 66); survivorship was more common in Australia (28.7%, *n* = 52), Canada (29.2%, *n* = 50), and the Netherlands (46.7%, *n* = 63); and Canada published the most on screening (45.6%, *n* = 78) (Figure 2). International collaborations made up 3.5% (*n* = 99) of the publications (Table 1). Across all countries, research on prevention, risk assessment, and treatment had fewer outputs (Figure 2).

Colorectal (*n* = 489, 17.5%), breast (*n* = 362, 12.9%), cervical (*n* = 227, 8.1%), prostate (*n* = 163, 5.8%), and lung (*n* = 151, 5.4%) represented the top five cancer types investigated. Overall, 31.5% (*n* = 880) of included studies investigated cancer in general or multiple cancer types (see Supplementary Table S2).

Observational studies were the most common study design (*n* = 1523, 54.4%), consisting primarily of cross-sectional and cohort studies (Table 2). Qualitative research represented 13.0% (*n* = 365) of studies, while interventional studies only accounted for 10.5% (*n* = 294). Of these, only 7.3% (*n* = 205) included studies were randomised controlled trials (RCTs). Nearly 60% of RCTs were about screening (58.5%, *n* = 120), followed by survivorship (13.2%, *n* = 27) and diagnosis (12.7%, *n* = 26) (data not shown).

**Table 2.** Distribution of study designs across included publications

**Study designs**	***n* (%)**
**Unfiltered evidence**	**2631 (94.0)**

**Observational**	**1523 (54.4)**

Cross-sectional	698 (45.8)
Cohort	656 (43.1)
Case-control	100 (6.6)
Observational (non-descript)	48 (3.2)
Ecological	12 (0.8)
Before and after	9 (0.6)
Total	1523 (100)

**Interventional**	**294 (10.5)**

Randomised controlled trial	205 (69.7)
Pilot	44 (15.0)
Feasibility	33 (11.2)
Non-randomised trial	12 (4.1)
Total	294 (100)

**Other**	**814 (29.1)**

Qualitative	365 (44.8)
Audit	150 (18.4)
Evaluation	123 (15.1)
Mixed methods	87 (10.7)
Other	69 (8.5)
Implementation studies	20 (2.5)
Total	814 (100)

**Filtered evidence**	**167 (6.0)**

Systematic reviews	127 (76.0)
Guidelines	40 (24.0)
Total	167 (100)

**Grand total**	**2798 (100)**

There were differences in the sex balance between first and last authors. Female authors represented 66.5% (*n* = 1304/1960) of first authors, but only 47.0% (*n* = 927/1971) of last authors. Sex was unable to be identified for 4% of first, and 2.8% of last authors. Additionally, 15 publications only had a single author (data not shown).

There was a mean of 400 (standard deviation [SD] 86.3) cancer in general practice publications each year. The mean percentage change in publications per year was 0.8% (SD 17.8) (Table 3).

**Table 3.** Cancer in primary care publications per year (2013–2019)

**Year**	**Publications, *n* (%)**	**% change/year**
2013	352 (12.6)	—
2014	404 (14.4)	14.8
2015	379 (13.5)	−6.2
2016	396 (14.2)	4.5
2017	432 (15.4)	9.1
2018	421 (15.0)	−2.5
2019	414 (14.8)	−1.7
**Total**	**2798 (100)**	

## DISCUSSION

### Summary

It is believed that this is the first article to explore the breadth and depth of cancer in general practice research using bibliometric analysis. The results suggest cancer in general practice is a stable field where research is predominantly descriptive or observational, and where there is less evidence outlining implementation or translation into clinical practice. This could suggest not only that there remains a paucity of clinical trial evidence, but also that further emphasis on interventional and/or implementation research is still needed. A wide geographical variation was also found in the focus of cancer in general practice research, and certain tumour types that are over-represented compared with their population disease burden.

A fundamental goal with general practice research is to develop evidence to inform clinical practice and improve outcomes for patients. The results suggest that most of the published cancer in general practice literature sits early in the research translation pipeline (that is, research that is still working towards understanding a problem or clinical need rather than testing an intervention to address this problem).^16^ The smaller number of RCTs and implementation studies suggests greater support is needed for so-called T2 and T3 translational studies where evidence is applied in practice.^16^ It further highlights the long trajectory to integrate evidence into clinical recommendations^17^ and the need to provide support to improve research translation. In Australia, only 18% of cancer research funding is awarded to studies that have moved beyond early translation.^18^

Cancer in general practice research is published in a wide range of journals, with only three of the top 10 journals being primary care specific, suggesting researchers target general medical and cancer-specific journals in which to share their findings. This may reflect a pattern of researchers chasing higher impact factor journals first, which are more likely to be in these broader areas than primary care- specific ones.^19^ More than two-thirds of publications came from journals with an impact factor of <5, suggesting that leading primary care journals have lower impact factors and/or that it may be difficult to publish cancer in general practice research in higher impact factor general medical and cancer-specific journals. A 2007 bibliometric analysis of cancer research in medical literature found that 25% of publications were published in the top 20 medical journals with the highest impact factors.^10^ The top 10 medical journals had an average impact factor of 30.3, which is nearly 10 times higher than that found in the present study.

### Strengths and limitations

The search strategy was designed to retrieve many publications relevant to cancer in primary care. Given the complexity of the structure of primary care and general practice between different countries, it was chosen to focus the bibliometric review on general practice only. This limits the applicability of results to this setting rather than primary care more broadly. This complexity is also a limitation to identification and interpretation of international collaborations in cancer in general practice research. Additionally, there is a dearth of relevant literature to compare the search strategies employed by other studies.

The authors tried to mitigate the variation in general practice nomenclature between countries by being inclusive around language used to describe both primary care and primary healthcare professionals. Not all general practice research is accurately labelled or signposted in indexing applied by the databases used for this search. This is particularly important when considering prevention research where the link to cancer may be less explicitly stated and result in an artificially lowered publication count.

This analysis represents research over a 7-year period recorded in two key databases. The analysis only applied to indexed journals and consequently does not cover unpublished research, works in non-indexed journals, and non-journal outputs such as books, theses, reports, or government documents. Given the English language restriction, this study does not capture all cancer in general practice publications internationally. Differences in healthcare systems between countries created an additional layer of complexity in identifying whether research was conducted in, or significantly related to, general practice. Overall, the results of this study illustrate the difficulty in accurately assessing research publications across areas of the cancer continuum based on the complexity and variation in the structure of general practice in different countries.

Some journals publish authors with first name initials only. The research team tried to identify the correct researcher based on authorship, but this was difficult in some cases. Additionally, information about impact factors and the appropriate figure at time of publication was not always available.

Lastly, impact factors remain a contentious measure of impact and have known limitations such as the length of citation windows, differences between disciplines, and the impact of commentaries and editorials artificially inflating calculations.^20^ Alternative metrics are increasingly being used to assess impact. Given the varied approaches to journals incorporating alternative metrics, in this context, impact factor was a more widely available variable to collect.

### Comparison with existing literature

Overall, cancer screening, diagnosis, and survivorship were the most investigated areas, although coverage across the cancer continuum varied considerably by country. This may highlight the different research priorities and targeted funding within countries. For example, from 2006–2011, Australia and the UK had similar levels of investment into cancer research when measured per head of population and as a percentage of the gross domestic product.^21^ In 2014, Cancer Research UK, the world’s largest independent cancer research charity, began substantially investing in early detection research through their national Early Diagnosis Initiative. This includes the International Alliance for Cancer Early Detection, which is a £55 million partnership between Cancer Research UK and the US.^22^ This initiative was partly in response to poorer survival outcomes for UK patients with cancer when compared with countries such as Australia, Canada, and Sweden, which was demonstrated through the International Cancer Benchmarking Partnership.^23^ While the pattern of research investment across the cancer continuum has been similar between the UK and Australia,^21^ key organisations, such as the Cancer Council Australia, have invested more in survivorship research. Cancer Council Australia is the largest non-government funder of cancer research in Australia.^24^ In 2020, they invested AUD$54 million into cancer research, over 70% of which was awarded to support and survivorship research.^24^

The distribution of publication by cancer type also highlights the well-known mismatch between disease burden and funding allocation.^25^^,^^26^ Publication outputs can be used as a proxy measure for funding and investment.^26^ In Australia, the cancer type with the greatest difference between incidence and mortality is liver cancer.^27^ It is the 15th most common cancer type but represents the eighth most common cause of cancer-related deaths.^27^ Only three Australian publications were found to be investigating liver cancer.^28^^–^^30^ However, the proportion of lung cancer publications identified in this study is comparable with current broader lung cancer research, representing only 6.5% of cancer research.^31^ This small percentage does not align with the global burden of lung cancer, as the second most commonly diagnosed cancer and the leading cause of cancer death.^1^ A 2020 bibliometric analysis of lung cancer research worldwide identified that almost all countries, except for China, have seen a decline in lung cancer research outputs over the past 15 years.^31^ In Australia, lung cancer is the greatest contributor of cancer disease burden but receives less research funding than colorectal, breast, prostate, melanoma, leukaemia, brain, ovarian, and liver cancer.^18^ The present study revealed that at least twice as many publications were about either breast or colorectal cancer in comparison with lung cancer. The higher frequency of these publications may be owing to the strong existing role of general practice in the care of patients with these cancers. For example, many countries have population- based colorectal cancer screening where general practice involvement has been widely researched.^32^^–^^35^ Additionally, the prevalence of breast and colorectal cancer publications aligns with that seen in the wider cancer literature, both of which are in the top three cancer types published.^36^ As countries consider future models of implementing lung cancer screening, it is possible that this pattern of research activity in general practice may change.

Overall, the variation in distribution of research publications by cancer type suggests that this inequity in cancer research funding may also be reflected in the support provided to primary care-based research.^25^^,^^37^ The potential mismatch in research funding needs to be considered in the context of variation of disease prevalence and economic standing between countries.^38^ These two lenses, along with the structure of the local health system, all likely play a role in the geographical variation seen in publication outputs by cancer type and area of the continuum.

### Implications for research and practice

The findings suggest that a large proportion of cancer in general practice research is observational. There is a need to provide additional support to assist researchers to use this evidence to underpin an increase in the development of interventional research. Additionally, the results suggest that certain tumour types are over-represented in the cancer in general practice literature compared with their population disease burden. Future consideration should be given on how to increase research of under- represented areas including both specific tumour types and areas of the cancer continuum such as cancer prevention.

There are a number of aspects of medical research that demonstrate sex inequality, such as research grant applications,^39^ and authorship of publications where, depending on the research field, females are significantly less likely to occupy first or last author positions.^40^^–^^42^ The results suggest this inequality may not extend to authors working in cancer in general practice research, and, in fact, female authors were found to represent two-thirds of first authors. This result is in line with a bibliometric study in primary health care and general internal medicine that found 63% of first authors in primary healthcare journals were female.^43^

International collaborations appear to be limited and consideration of how to better enable the development of large-scale, multi-country studies may be valuable. This is also affected by the known limited capacity of low- and middle-income countries to undertake primary healthcare research.^44^

In conclusion, it is believed this is the first study to explore cancer in general practice research using bibliometric analysis. The study provides an interesting insight into the breadth of cancer in general practice research. It offers a concise view of the type of research conducted, where it was conducted, and the different research focus between different countries. It could also be used for future priority-setting exercises by funders and researchers to determine where best to invest limited resources for primary care cancer research.
